# Comparative transcriptome analysis of genes involved in paradormant bud release response in ‘Summer Black’ grape

**DOI:** 10.3389/fpls.2023.1236141

**Published:** 2023-09-25

**Authors:** Shaogang Fan, Feixiong Luo, Meijun Wang, Yanshuai Xu, Wenting Chen, Guoshun Yang

**Affiliations:** College of Horticulture, Hunan Agricultural University, Changsha, Hunan, China

**Keywords:** auxin, endodormancy, paradormancy, second crop cultivation, short pruning, transcriptome

## Abstract

Grapevines possess a hierarchy of buds, and the fruitful winter bud forms the foundation of the two-crop-a-year cultivation system, yielding biannual harvests. Throughout its developmental stages, the winter bud sequentially undergoes paradormancy, endodormancy, and ecodormancy to ensure survival in challenging environmental conditions. Releasing the endodormancy of winter bud results in the first crop yield, while breaking the paradormancy of winter bud allows for the second crop harvest. Hydrogen cyanamide serves as an agent to break endodormancy, which counteracting the inhibitory effects of ABA, while H_2_O_2_ and ethylene function as signaling molecules in the process of endodormancy release. In the context of breaking paradormancy, common agronomic practices include short pruning and hydrogen cyanamide treatment. However, the mechanism of hydrogen cyanamide contributes to this process remains unknown. This study confirms that hydrogen cyanamide treatment significantly improved both the speed and uniformity of bud sprouting, while short pruning proved to be an effective method for releasing paradormancy until August. This observation highlights the role of apical dominance as a primary inhibitory factor in suppressing the sprouting of paradormant winter bud. Comparative transcriptome analysis revealed that the sixth node winter bud convert to apical tissue following short pruning and established a polar auxin transport canal through the upregulated expression of *VvPIN3* and *VvTIR1*. Moreover, short pruning induced the generation of reactive oxygen species, and wounding, ethylene, and H_2_O_2_ collectively acted as stimulating signals and amplified effects through the MAPK cascade. In contrast, hydrogen cyanamide treatment directly disrupted mitochondrial function, resulting in ROS production and an extended efficacy of the growth hormone signaling pathway induction.

## Introduction

The grapevine shoot system originates from a hierarchy of buds in the leaf axil. The axillary bud (N+1) or prompt bud grows during the current season and becomes the lateral shoot. The latent bud (N+2) or winter bud is located at the base of the lateral shoot, which may form the inflorescence primordia and usually spans two years, bursting in the next spring and becoming the main shoot (N), which gives rise to the next season’s crops (Pratt, 1974; Lavee and May, 1997). The winter bud undergoes three well-defined stages of development: the formation of anlagen, the formation of inflorescence primordia, and the formation of flowers (Srinivasan and Mullins, 1976). The first two stages are completed during the growing season of the first year, while the final stage occurs during the second year. Fully developed winter bud contain one or more inflorescence primordia with many branch primordia, resembling a cluster of grapes (Noyce et al., 2015). Flower formation occurs shortly before and during bud burst in the next spring while the vines undergo a period of temporary growth cessation known as dormancy in winter.

Bud dormancy was defined as “the temporary cessation of visible growth of meristems,” which enables plant survival under adverse environmental conditions and categorized dormancy into three types: paradormancy, endodormancy, and ecodormancy (Lang et al., 1987; Horvath et al., 2003; Singh et al., 2017). Growth cessation is regulated by the *CONSTANS* (*CO*)/*FLOWERING LOCUS T* (*FT*) regulatory module (Bohlenius et al., 2006). Short days (SDs) signals are mediated by the active expression of *LATE ELONGATED HYPOCOTYL 2* (*LHY2*), which represses the *FT2* expression in poplar (Hsu et al., 2011; Ramos-Sanchez et al., 2019). *FT2* interacts with the bZIP transcription factors *FD-like 1* (*FDL1*) to form the FDL1–FT complex that downregulates the downstream target gene *APETALA1-like-* (*LAP1*) (Tylewicz et al., 2015). SDs induce down regulation of the *AINTEGUMENTALIKE* (*AIL1*) gene and up regulation of the *BRANCHED1* (*BRC1*) gene, both of which are modulated by *LAP1. AIL1* acts on the promoter region of cyclin genes, such as *CYCD3:2*, to positively regulate cell-cycle gene expression, while BRC1 physically interacts with FT2 and counteracts its function, resulting in the inhibition of growth (Karlberg et al., 2011; Azeez et al., 2014; Maurya et al., 2020).

Endodormancy differs from growth cessation in that it is regulated through abscisic acid (ABA) acting on intercellular communication (Tylewicz et al., 2018). SDs induce an increase in ABA levels. ABA suppresses *PICKLE* (*PKL*) to induce *SHORT VEGETATIVE PHASE-LIKE* (*SVL*) expression, forming a regulatory network that positively regulates *SVL* (Singh et al., 2018; Singh et al., 2019). *SVL* then induces the upregulation of *CALLOSE SYNTHASE 1* (*CALS1*) expression, which leads to callose formation and blocks intercellular communication via plasmodesmata blockage, thereby interrupting growth-promoting signals from the meristem (Singh et al., 2019). Numerous studies have been conducted to reveal the mechanism underlying grapevine endodormancy release. Hydrogen peroxide (H_2_O_2_) is the key signal for grape bud endodormancy release, and both natural low-temperature chilling treatment and chemical stimuli, e. g. hydrogen cyanamide (HC) treatment, inhibit catalase (CAT) activity, leading to an accumulation of H_2_O_2_ in dormant bud, which induces bud break (Nir et al., 1986; Or et al., 2002; Pérez et al., 2008). HC treatment inhibits mitochondrial O_2_ uptake and induces respiratory and oxidative stress, leading to the upregulation of calcium sensors involved in the release of grape bud dormancy (Keilin et al., 2007; Pang et al., 2007; Ophir et al., 2009; Perez et al., 2009). Moreover, HC treatment results in a decrease in ABA content and the removal of ABA-mediated repression by down-regulating the transcription levels of ABA synthesis-related genes *VvXERICO* and *9-cis-epoxycarotenoid dioxygenase* (*VvNCED*), while significantly upregulating the expression of the ABA 8′-hydroxylase gene (*VvA8H-CYP707A4*), an ABA degradation enzyme (Zheng et al., 2015; Zheng et al., 2018a). HC treatment also upregulates the expression of specific 1-aminocyclopropane1-carboxylic acid (ACC) synthase (*VvACS*) and ACC oxidase (*VvACO*) genes, leading to increased biosynthesis of ethylene (Shi et al., 2018). Ethylene signaling targets, ethylene responding factors (VvERF-VIIs), which in turn activate catabolism and meristem regrowth (Shi et al., 2020). Gibberellins (GAs) initially inhibit bud break initiation but subsequently promote meristem regrowth. HC treatment induces up-regulation of *GA2ox*, which acts as bioactive GAs deactivating enzyme, while down-regulating VvGA3ox and VvGA20ox, which convert inactive GA_12_ to bioactive GAs, GA_1_ and GA_4_. This results in a decrease in GA_1_ content and the elimination of GA’s negative effects on bud break (Zheng et al., 2018b).

Paradormancy, also referred to as apical dominance and described as bud “idling” state, is primary regulated by auxin and strigolactones (SLs) (Brewer et al., 2015; Waters et al., 2017; Wang et al., 2018). The process of lateral bud outgrowth under decapitation treatment in favorable environmental conditions can be divided into two phases: “activation” and “growth.” These phases can be explained by the nutritive hypothesis and the canalization hypothesis, respectively (Domagalska and Leyser, 2011; Waters et al., 2017; Wang et al., 2018). The shoot apical meristem (SAM) acts as a strong sink, competing with axillary buds for sugar and inhibiting their outgrowth through the action of *BRC1* (Aguilar-Martinez et al., 2007; Martin-Trillo et al., 2011; González-Grandío et al., 2013). Simultaneously, decapitation leads to auxin efflux from axillary buds through PIN-FORMED1 (PIN) proteins and establishes its polar auxin transport canal, which promotes vascularization to sustain growth (Barbier et al., 2019). Generally, sugar response is faster than indole-3-acetic acid (IAA) signals (Mason et al., 2014)*. BRC1* plays a central role in the hormone signaling pathway and inhibits bud release and branching outgrowth, bud not necessary in *Arabidopsis* (Seale et al., 2017). *BRC1* expression is negatively regulated by phytochrome B, and SLs promote *BRC1* expression, while cytokinins inhibit it (Dun et al., 2012; González-Grandío et al., 2013). BRC1 directly bind to *PIN3*, repressing axillary bud outgrowth in cucumber (Shen et al., 2019). BRC1 is also involved in inducing the expression of *NCED3*, resulting in ABA accumulation, which in turn suppresses bud growth (Gonzalez-Grandio et al., 2017).

Due to the widespread adoption of rain-shelter cultivation facilities and two-crop-a-year cultivation system, Southern China has become a major production region for Table grapes (Chen et al., 2017; Cheng et al., 2019). The second crop cultivation is achieved by forcing the paradormant buds of grapevines with fully developed inflorescence primordia to break dormancy between June and August, about a month after the first crop harvest, which yield a winter crop of better quality (Cheng et al., 2019). Short pruning and hydrogen cyanamide treatment are widely used as agronomic practices to release paradormancy and promote buds SAM regrowth (Chen et al., 2017). However, in second crop cultivation, winter bud should be in a paradormant state rather than endodormant, and decapitation can theoretically release the inhibition on grape buds. while HC is frequently used as an endodormancy-breaking agent for horticultural crops in warm-winter regions, its use is associated with significant drawbacks. HC treatment is commonly believed to promote bud sprouting and make the sprouting more uniform, but the mechanism behind it is relatively unknown. Additionally, HC reagents are highly phytotoxic and can cause bud damage (Or et al., 1999). High temperatures can also exacerbate its harmful effects on buds, and toxic exposure is particularly concerning when HC is applied during summer practices (Sharif and Fayed, 2021). Therefore, studying the mechanism of paradormancy release induced by HC can be beneficial for facilitating the search for low-toxicity and high-efficiency alternatives and improving cultivation techniques. In this study, we compared paradormancy and endodormancy characteristics in grapevine cv. ‘Summer Black’. We aimed to explore the regulatory mechanism of genes involved in HC-induced paradormancy release and compare it with HC-induced endodormancy release using transcriptomics technology.

## Materials and methods

### Plant materials

All the experiments were conducted at the ‘Gan Shan’ grapevine experimental vineyard of Hunan Agricultural University (Changsha, China) (N28°08′, E113°10′), between 2018 and 2021. The study utilized ten-year-old ‘Summer Black’ Table grape vines (*V. vinifera × V. labrusca*), grafted onto Beta rootstock. The vines were planted in a south-north orientation with a row spacing of 3.0 m (between vines) × 1.8 m (between rows), in soil composed of paddy soil amended with organic fertilizer. The cultivation was carried out under rain-shelter conditions, and a V-shaped horizontal shoot positioning system was implemented for training. The cordons were positioned at a height of 1.0 m above the ground, and each tree retained 30 shoots as fruiting branches. The spacing among the one-year young shoots was maintained at 20 cm using ropes. In the subsequent experiments, results were assessed based on shoot counts rather than vine counts. Meteorological data were obtained from a weather station within the vineyard.

The two-crop-a-year cultivation system encompasses distinct modes referred to as nonoverlap and overlap systems within subtropical regions (Chen et al., 2017; Cheng et al., 2019). In this research, the experimental grapevines were subjected to an overlap cultivation system. Conventionally, the growing season for the first crop (summer crop) spans from March to July, succeeded by the emergence of the second crop (winter crop) from August to November. However, these trials experienced modifications due to the pruning treatments applied. Viticultural practices were conducted following standard cultural practices commonly employed in commercial vineyards. The regime for the first crop encompassed winter pruning in January, retaining 1 to 2 buds. Subsequently, these buds underwent sprouting in mid-March. The emerging shoots were subjected to pinching just above the seventh nodes, a process executed around 30 days after bud break (DABB). Additionally, the basal lateral shoots (in positions 1-4) underwent trimming above the basal leaf, whereas the uppermost lateral shoot (located in positions 5-7) retained 4 leaves. Each sustained fruiting branch was endowed with a solitary cluster. Regarding the second crop, the practices of green shoot pruning and lateral shoot pruning were executed at the sixth node, commencing in mid-May. This strategic action facilitated the sprouting of the winter bud at the designated node, thereby promoting the development of winter bud lateral shoots. Typically, these shoots bear flower clusters, which are then harnessed for the cultivation of the second crop. In this scenario, each fruiting branch retained a solitary cluster, and the upper limit for the number of grains per cluster was upheld at 60.

### Single-node (bud) cutting

The grape bud-break response in single-node cuttings is highly correlated with bud behavior on the vine, making it a widely used and reliable indicator for evaluating the dormancy status of grapevines under forcing conditions (Camargo Alvarez et al., 2018). The depth of endormancy in grapevine winter bud was described by the bud break response of single-bud cuttings under forcing conditions, following the methods described in previous studies (Or et al., 2002; Shi et al., 2018). Detached canes with ten buds (in positions 3-12) were collected at two-week intervals between November 5th, 2018 and January 14th, 2019. On each collection date, nine groups of 10 single-node cuttings were prepared and inserted into containers filled with tap water. These containers were placed in a growth chamber at a temperature of 25°C under a 15 h/9 h light/dark regime (Perez and Lira, 2005). The appearance of green tissue under the bud scales was considered the criterion for bud break at the sixth node (Or et al., 2002).

Additionally, a modified method was employed to describe paradormancy characteristics and the correlative inhibition within the shoot system. Shoots were pruned from the base node in the vineyard, and detached prunings carrying seven buds (in positions 1-7) were transferred to the laboratory on May 20, 2019 (60 DABB). Single-node cuttings of the same node position were gathered, mixed, and divided into nine groups, each consisting of 10 cuttings. The cuttings were sterilized with 1% carbendazim for 30 minutes to prevent dieback, secured onto foam material plates, and placed inside square basins filled with tap water. The basins were placed in the growth chamber described above. The sprouting of winter bud was observed daily using the same criterion.

### Short prune and hydrogen cyanamide treatment

Shoots with consistent growth were selected and then randomly assigned to several groups. Shoots underwent short pruning (P) at the sixth node on May 15, May 25, June 10, and July 10, 2020, respectively. On August 10, 2020, short pruning and defoliation (PD) were carried out (**Table 1**). Each group consisted of 10 shoots, and there were 9 replicates. The sprouting of sixth node winter bud was observed and recorded.

**Table 1:** Short prune and hydrogen cyanamide treatment for inducing paradormancy release.

Code	Date	Treatment	Control
Short prune treatments			
1	May 15, 2020	P	Mock
2	June 10, 2020	P	Mock
3	July 10, 2020	P	Mock
4	August 10, 2020	PD	P
Hydrogen cyanamide treatment			
5	June 10, 2020	PHC	P
6	July 10, 2020	PHC	P
7	August 10, 2020	PHC	P
8	August 10, 2021	PDHC	PD
9	August 13, 2021	PDHC	PD

Hydrogen cyanamide (HC) treatments were divided into two categories, with leaves either retained or removed (**Table 1**). Prior to each experiment, the shoots were pruned above the sixth node and then randomly divided into treatment and control groups. The hydrogen cyanamide treatment group used a 2% hydrogen cyanamide (Darong, Ningxia, China) with 0.1% Tween 20 as a nonionic surfactant, while the control group used distilled water with 0.1% Tween 20, applied to the sixth node position winter bud. Each group consisted of 10 shoots, with a total of 9 replicates for each treatment and control. The experimental area was irrigated immediately after treatment to keep the soil moist. All experiments observed and recorded the results of bud break at the sixth node. On August 10, 2020, one group was destined for bud-break analysis, the other group was used to sample at 0, 24, 48, and 96 h after PDHC treatment for transcriptome analysis. Additionally, another the same experiment as Code 9 was carried out at August 8, 2021, for sampled buds of PDHC and PD at 4 h, 8 h, 24 h, 48 h, 96 h, and 192 h for expression analysis by qRT-PCR.

### RNA extraction and quantitative real-time PCR

Total RNA was extracted from buds that were quick-frozen in liquid nitrogen using plant RNA purification reagent for plant tissue according to the manufacturer’s instructions (Invitrogen, Carlsbad, CA, USA) and genomic DNA was removed using DNase I (TaKara, Dalian, China). Then RNA quality was determined by 2100 Bioanalyser (Agilent) and quantified using the NanoDrop 2000 (Thermo Scientific, Waltham, MA, USA), and the high-quality grape RNA samples was used to construct sequencing library.

To validate the RNA-Seq results and further investigate the gene expression changes between the PDHC treatment group and the PD group, quantitative real-time polymerase chain reaction (qRT-PCR) was performed on 17 selected genes. The Actin gene was used as a reference gene, and the primers utilized for these analyses are provided in **Table S1**. First-strand cDNA was synthesized from 1 µg of total RNA using the cDNA Synthesis SuperMix kit (Transgen, Beijing, China). All qRT-PCR reactions were conducted on a CFX96 Real-Time PCR instrument (Bio-Rad, Hercules, CA, USA). Each reaction was performed in triplicate with a total volume of 10 µL, containing 5 µl of 2× TransStart® Tip Green qPCR SuperMix (Transgen), 0.5 µl of each primer (10 µM), 1 µl of cDNA, and 3 µl of nuclease-free water. The PCR program started with an initial denaturation step at 95°C for 30 s, followed by 40 cycles of 95°C for 5 s and 60°C for 30 s. The relative gene expression levels were calculated using the 2^(-ΔΔCT)^ method. Statistical analyses were performed using SPSS 18.0 software (Chicago, IL, USA).

### Transcriptome analysis

12 RNA-seq transcriptome libraries were prepared using the TruSeq™ RNA sample preparation Kit for Illumina (San Diego, CA, USA), with 1μg of total RNA for each sample. The libraries were sequenced using the Illumina HiSeq XTen system at the Shanghai Majorbio Bio-pharm Biotechnology Co. (Shanghai, China). After removing adapters and discarding low-quality sequences, the clean reads were aligned to the reference genome of *Vitis vinifera* Assembly (12X.2) and its annotation (VCost.v3) (https://urgi.versailles.inra.fr/Species/Vitis/Annotations) (Canaguier et al., 2017). The quality of the sequences was assessed through saturation and gene coverage analyses using RSeQC-2.3.6 software.

The expression level of each transcript was calculated using the Kilobase of transcript per Million mapped reads (FPKM) method. Genes with FPKM < 1 in all samples were considered invalid and excluded from the analysis. Three sets of differentially expressed genes (DEGs) were identified by comparing the counts at 24 h, 48 h, and 96 h with 0 h using the criteria of p-adjust < 0.05 and |log2FC|≥ 1. GO functional enrichment analysis and KEGG pathway analysis were conducted using GOAT tools and the R package, respectively (Klopfenstein et al., 2018). The gene expression trend analysis were analyzed using maSigPro (Conesa et al., 2006).

## Results

### Characteristics of dormancy in ‘Summer Black’ grape winter bud

**Figure 1:**
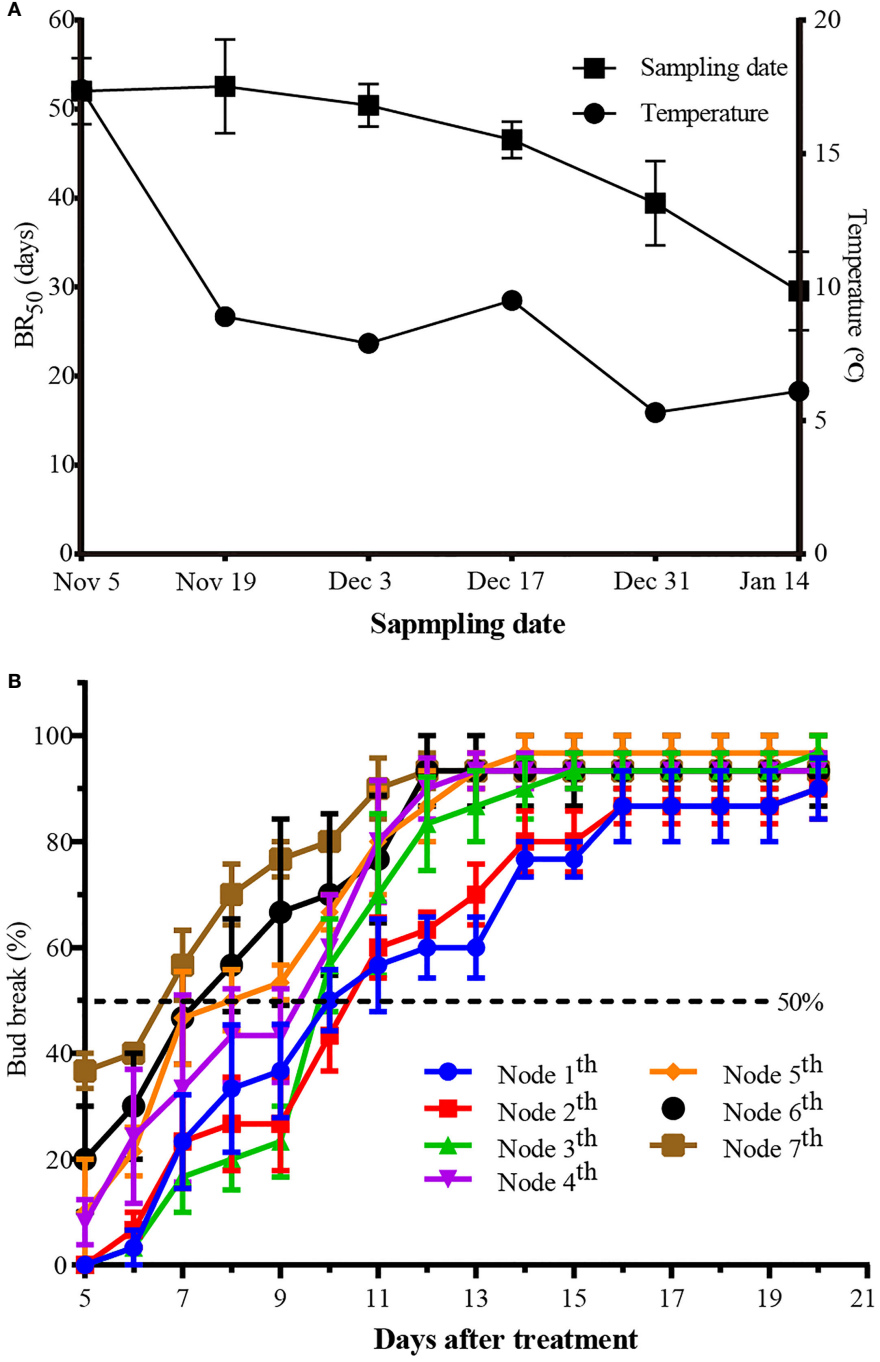
Bud outgrowth potential of single-node cutting. **(A)** Days required for 50% bud break during endodormancy. Singel node cutting were collected during Nov 5, 2018 to Jan 14, 2019, the right-hand Y-axis represents the daily mean temperature. **(B)** Days required for 50% bud break during paradormancy, Singel node cutting were collected at May 20, 2019. BR_50_ indicate the mean time required for reaching 50% of bud-break under forcing conditions. Values are means of nine replicates with ten shoots each, the bar represents standard deviation.

The parameter BR_50_, which was defined as estimated time required to reach 50% bud break, has been used to describe and compare the depth of dormancy (Perez et al., 2007; Pérez et al., 2008). It was observed that the degree of endodormancy was the deepest in November in the Changsha region, with a BR_50_ value of more than 50 days. This value was shortened to around 30 days with the accumulation of chilling in mid-January (**Figure 1A**). While the paradormant buds burst from the fifth day since treatment, the top nodes (5-7 nodes) rapidly sprouted, with a BR_50_ of 7 days, while other nodes took almost 10 days (**Figure 1B**). It is apparent that the upper part of the same shoot was the least inhibited and most prone to sprout, while the lower part was the most inhibited and least likely to sprout.

### Short pruning affects the paradormancy release of grape winter bud

**Figure 2:**
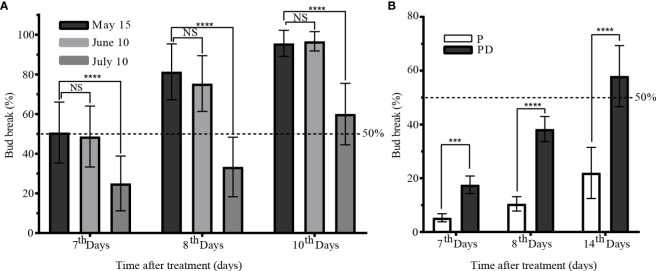
Characteristics of paradormant buds release by short pruning. **(A)** Bud break of pruning treatment at May 15, June 10, and July 10. **(B)** Comparison of bud break between the pruning treatment and pruning plus artificial defoliation on August 10. P: short prune; PD: prune plus artificial defoliation; Values are presented as means ± standard deviation of 15 replicates, with ten shoots each. The data were analyzed using Student′s two-tailed unpaired t-test (***, *p*<0.001; **** *p <*0.0001). NS, not significant.

From May to August, the winter bud were in a state of paradormancy. After short pruning (P) treatment before June 10, the sixth node winter bud showed a sprouting rate of 50% on the 7^th^ day. Similarly, after short pruning on July 10, the sprouting rate reached 60% on the 10^th^ day, while winter bud without short pruning cannot sprout (**Figure 2A**). The sprouting rate of winter bud after short pruning with retained leaves on August 10 was only 22% on the 14^th^ day. However, the sprouting rate increased to 58% after short pruning plus artificial defoliation (PD) treatment (**Figure 2B**). These findings suggest that the winter bud remained paradormant before August 10, and senescent leaves had an inhibitory effect on winter bud sprouting. Short pruning and defoliation were found to be effective methods for release paradormancy in winter bud.

### The effect of hydrogen cyanamide on bud paradormancy release

**Figure 3:**
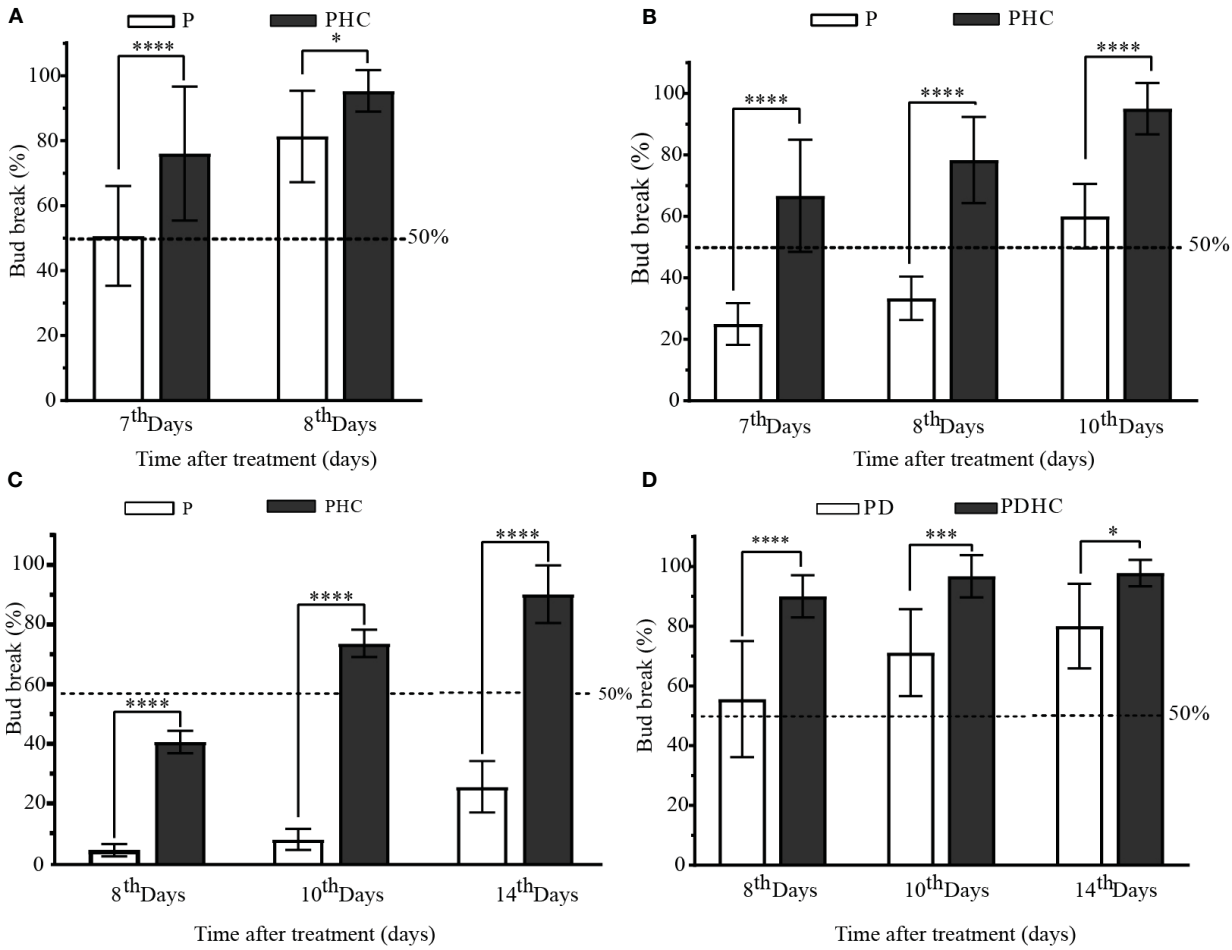
Characteristics of paradormant buds release by short pruning and hydrogen cyanamide treatment. **(A-D)** The sprouting rate of paradormant buds by short pruning, hydrogen cyanamide, and artificial defoliation treatment at June 10, July 10, and August 10 treatments. P: short prune; PHC: short prune plus hydrogen cyanamide treatment; PDHC: prune plus artificial defoliation plus hydrogen cyanamide treatment; Values are means of 15 replicates with ten shoots each, the bar means standard deviation. The data were analyzed using Student′s two-tailed unpaired t-test (*, *p*<0.05; ***, *p*<0.001; **** *p <*0.0001).

Compared to the single short pruning treatment, added HC (PHC) treatment significantly increased the sprouting rate of paradormant buds. On June 10, the sprouting rate of control group (P) reached nearly 80% on the 8^th^ day after short pruning treatment, while the PHC treatment resulted in almost 100% sprouting rate and was more uniform (**Figure 3A**). The effect of hydrogen cyanamide was more prominent in the late paradormancy release treatments. In the treatment on July 10, the sprouting rate of the P control group was only 20% on the 7^th^ day, while the PHC treatment group exceeded 60%, and the sprouting rate approached 100% on the 10^th^ day (**Figure 3B**). In the treatment on August 10, the sprouting rate of the PHC treatment group was nearly 64% on the 8^th^ day, while the P control group had only 7% (**Figure 3C**). The sprouting rate of the PD group exceeded 50% on the 8^th^ day, while added HC (PDHC) treatment group was nearly 90% (**Figure 3D**). These results indicate that the application of HC can significantly enhance the speed and uniformity of bud break.

### Analysis of differentially expressed genes in HC induced paradormant bud release

To investigate the molecular changes underlying PDHC-induced paradormancy release, a total of 12 transcriptome libraries were constructed, with three library repeats for each time point. The resulting high-quality clean reads were aligned to the grape reference genome database, with 87% of reads uniquely mapped and 2% of reads mapped to multiple locations (**Table S2**). Saturation curve analysis showed that most genes with FPKM > 3.5 reached saturation at approximately 65% mapped reads (**Figure S1**).

**Figure 4:**
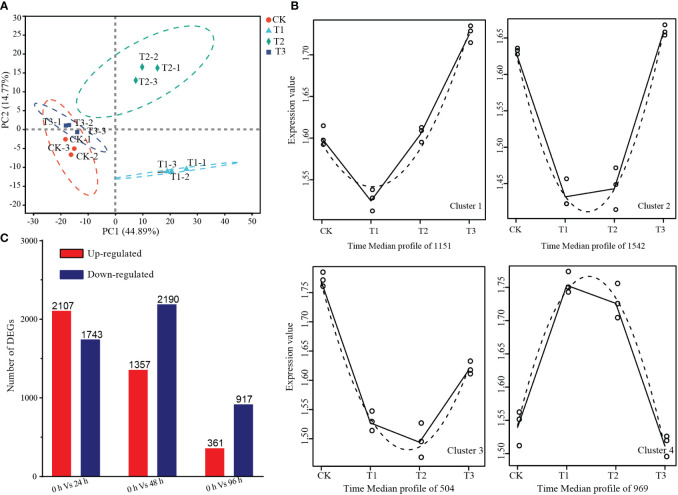
Summary of transcriptome analysis. **(A)** Principal component analysis of the expression levels between the samples. **(B)** Time-series differential expression analysis of gene expression changes over time revealed significant alterations in the expression levels of numerous genes in response to the treatment. **(C)** Number of differentially expressed genes at 24 h, 48 h, and 96 h of treatment.

The principal component analysis (PCA) revealed that replicates for the same time point were closely clustered together, indicating good reproducibility. The samples were separated according to the time points on the PC1, with a variation of 44.89%, suggesting that the samples exhibited time-dependent changes during the dormancy-breaking treatment (**Figure 4A**). The time-series differential expression analysis revealed there were 4166 high-level transcripts clustered into four distinct clusters. Among these clusters, 3197 genes exhibited a downregulation trend in gene expression 24 h after the dormancy-breaking treatment. Cluster 1 comprises 1151 genes that initially exhibited downregulation at 48 hours, displaying subsequent recovery and an upward trend. On the other hand, Cluster 4 encompasses 969 genes that were initially upregulated but subsequently reverted to their pre-treatment expression levels (**Figure 4B**). In total, 5736 differentially expressed genes were identified, with 2107, 1357, and 361 genes upregulated at 24 h, 48 h, and 96 h of treatment, respectively, and 1743, 2190, and 917 genes downregulated (**Figure 4C**).

**Figure 5:**
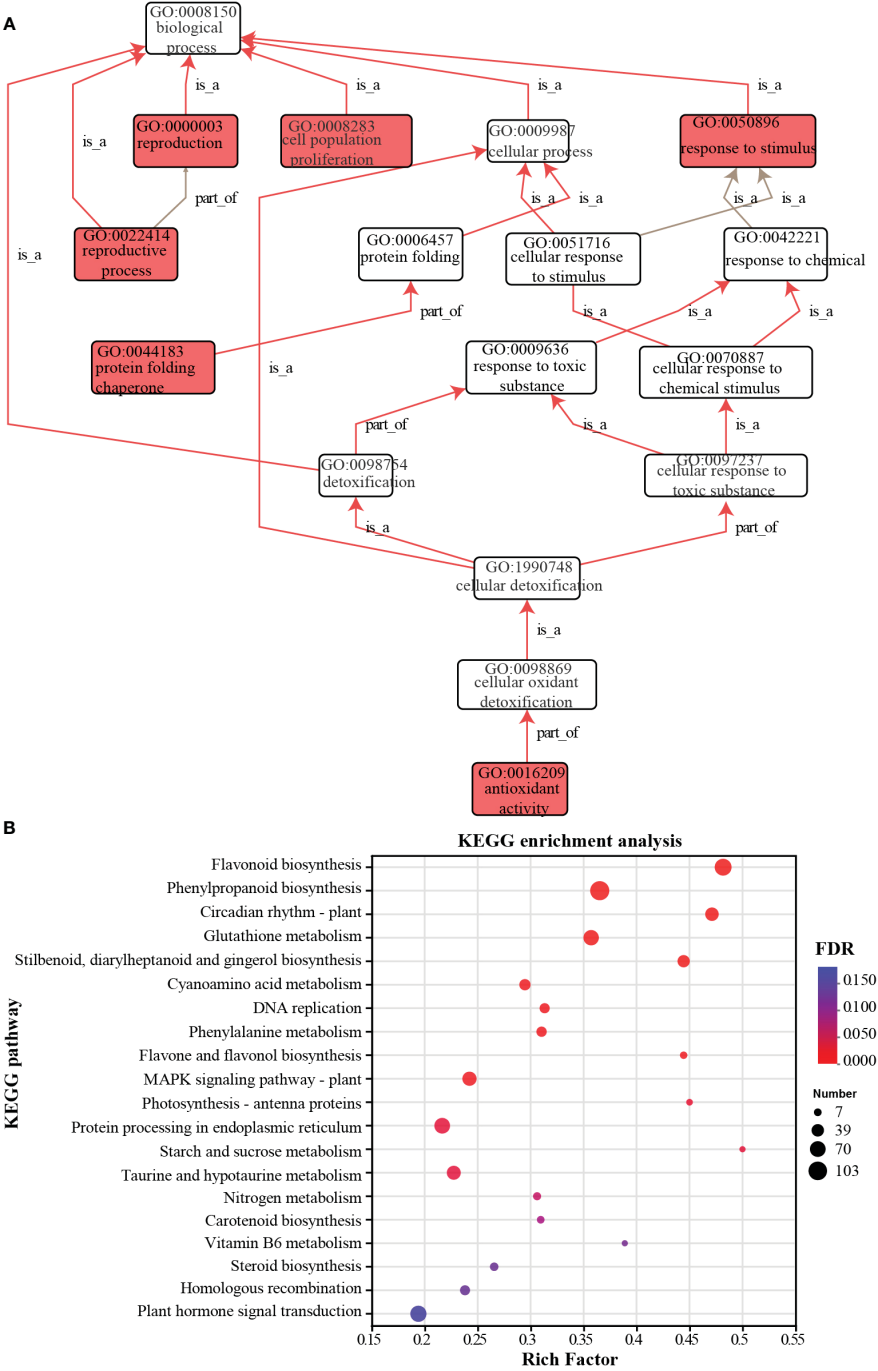
GO and KEGG enriched analysis of differentially expressed genes. **(A)** The directed acyclic graph displays the enriched GO terms resulting from the analysis of differentially expressed genes. **(B)** Significant bubble plot of KEGG enriched pathways analysis of differentially expressed genes. The circle size represents the number of DEGs detected in the KEGG pathway. The rich factor is the ratio of DEGs to the total background gene number in each pathway.

GO function enrichment analysis identified 170 terms in biological process, 90 terms in molecular function, 32 terms in cellular component with FDR < 0.05 (**Table S3**). The enriched GO terms related to biological processes were displayed in a directed acyclic graph (DAG) with p-value < 0.05, with terms such as reproduction, cell population proliferation, and response to stimulus highlighted in red (**Figure 5A**). KEGG enrichment analysis revealed a total of 127 pathways, among which 25 pathways showed significant enrichment with a *p*-value < 0.05 (**Figure 5B**; **Table S4**). Among these pathways, several pathways related to plant DNA replication and repair, such as mismatch repair, DNA replication, base excision repair, and homologous recombination pathways, were enriched and showed a downregulated expression trend (**Figure S2**). Additionally, pathways involved in plant stress response, such as glutathione metabolism, MAPK signaling pathway, and protein processing in endoplasmic reticulum, were also enriched (**Table S5**; **Figures S3**, **S4**). The activation of the glutathione peroxidase (GPX) cycle and the ascorbate-glutathione (AsA-GSH) cycle upregulates oxidative stress-related genes, including glutathione S-transferase (*VvGST*), L-ascorbate peroxidase (*VvAPX*), gamma-glutamyl transpeptidase 3 (*VvGGT3*), glutathione synthetase (*VvGSS*), and glutamate-cysteine ligase (*VvGCL*). Furthermore, other enriched pathways included the plant hormone signaling pathway, pathways involved in energy metabolism under stress, such as starch and sucrose metabolism, and pentose phosphate pathway.

### Transcript changes in signal transduction pathway-related genes during paradormant bud release

**Figure 6:**
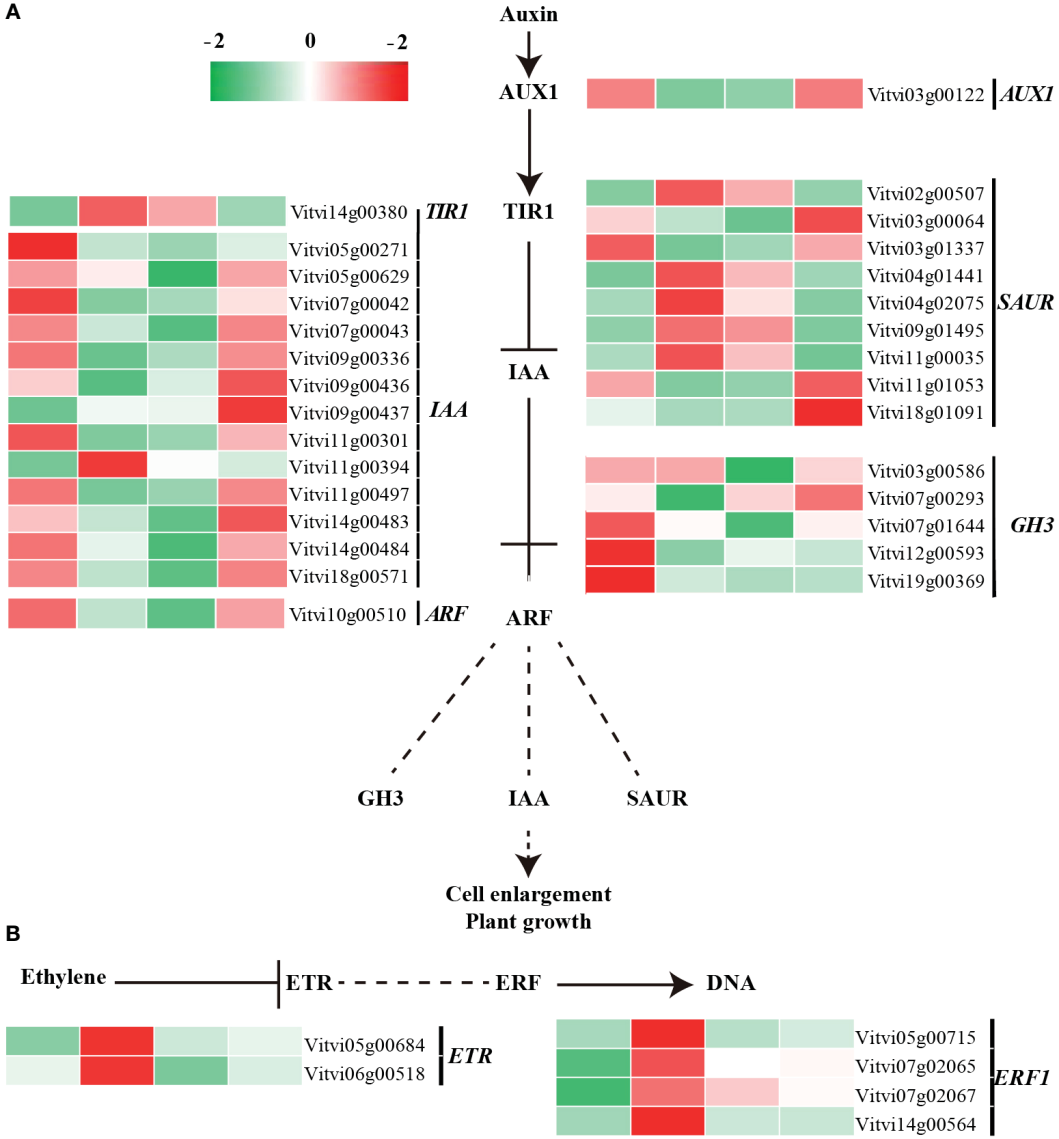
Transcript levels of auxin and ethylene signaling pathway-related genes during paradormant bud release. **(A)** Auxin and **(B)** ethylene signal transduction pathway.

Auxin signaling play a critical role in paradormancy release. Following decapitation treatment, the sixth node winter bud was transformed into biological apex. The coding sequence of auxin transporter-like protein 1 (*VvAUX1*) was downregulated after 24 h of treatment, while the coding sequence of auxin receptor, transport inhibitor response 1 (*VvTIR1*), was up regulated (Hayashi et al., 2008; Vandenbussche et al., 2010). The genes coding for the auxin-responsive proteins (*VvAux/IAA*), which function as transcriptional factors that repress auxin response, showed downregulation after treatment, with 12 out of 13 downregulated. Decreased abundance of Aux/IAA proteins may weaken the inhibitory effect on auxin signals by binding with auxin response factor (VvARF). The genes coding for indole-3-acetic acid-amido synthetase, *VvGH3*, were downregulated, while the auxin-responsive protein genes (*VvSAUR*), were upregulated after treatment (**Figure 6A**; **Table S5**). Consequently, the auxin signal was transmitted.

Moreover, wounding, ethylene, and H_2_O_2_ serve as stimulating signals enriched in the MAPK signaling pathway and collectively participate in paradormancy release. The ethylene signaling pathway, including genes coding for ethylene receptor 2 (*VvETR*) and ethylene-responsive transcription factor (*VvERF*), were upregulated after treatment (**Figure 6B**). Genes involved in ethylene synthesis pathways, represented by 1-aminocyclopropane-1-carboxylate oxidase 2 (*VvACO*), were upregulated and enriched in the cysteine and methionine metabolism pathway (**Table S6**). The ABA signaling pathway was also activated, as evidenced by the upregulation of four ABA receptor *VvPYL* genes and one serine/threonine-protein kinase gene (*SnRK2*), This suggests the completion of ABA signal transduction (**Figure S5**; **Table S6**). Additionally, the sequences coding for calcium-binding protein (*VvCaM*), cysteine-rich kinase (*VvCRK*), respiratory burst oxidase homolog protein (*VvRboh*), mitogen-activated protein kinase kinase (*VvMKK*), and mitogen-activated protein kinase (*VvMPK*) all exhibited significant upregulation after treatment (**Figure S4**).

### Transcript changes in starch and sucrose metabolism pathway-related genes during paradormant bud release

**Figure 7:**
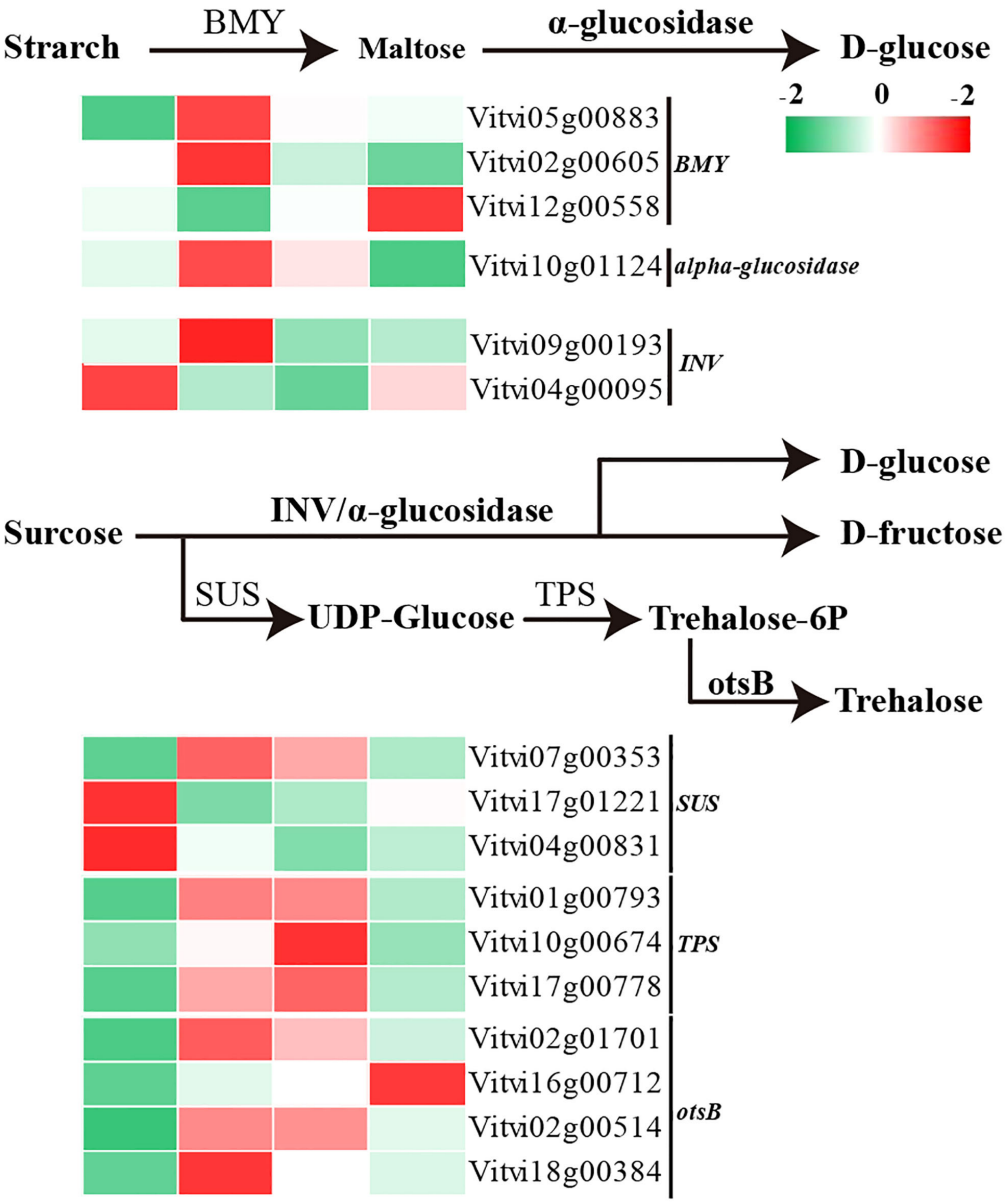
Transcript levels of starch and sucrose metabolism pathway-related genes during paradormant bud release.

Sugars play a dual role in providing both sugar signals and energy for the paradormancy release. Following decapitation treatment, the coding sequences for the β-amylase (*VvBMY*) and alpha-glucosidase are upregulated (**Figure 7**). These enzymes mediate the conversion and degradation of starch into glucose. The beta-fructofuranosidase gene (*VvINV*) is also upregulated along with alpha-glucosidase, and both enzymes are involved in the conversion of sucrose into glucose and D-fructose. In addition, trehalose synthesis is promoted through the upregulation of the sucrose synthase encoding gene (*VvSUS*), alpha-trehalose-phosphate synthase encoding gene (*VvTPS*), and trehalose-phosphate phosphatase encoding gene (*VvotsB*).

### Effects of hydrogen cyanamide on transcript levels of signal transduction pathway-related genes.

The reliability of the RNA-Seq data was confirmed by conducting qRT-PCR analysis. Linear regression analysis revealed a determination coefficient of 0.9228 between the RNA-Seq and qRT-PCR data, indicating a high level of concordance between the two methods (**Figure S6**).

**Figure 8:**
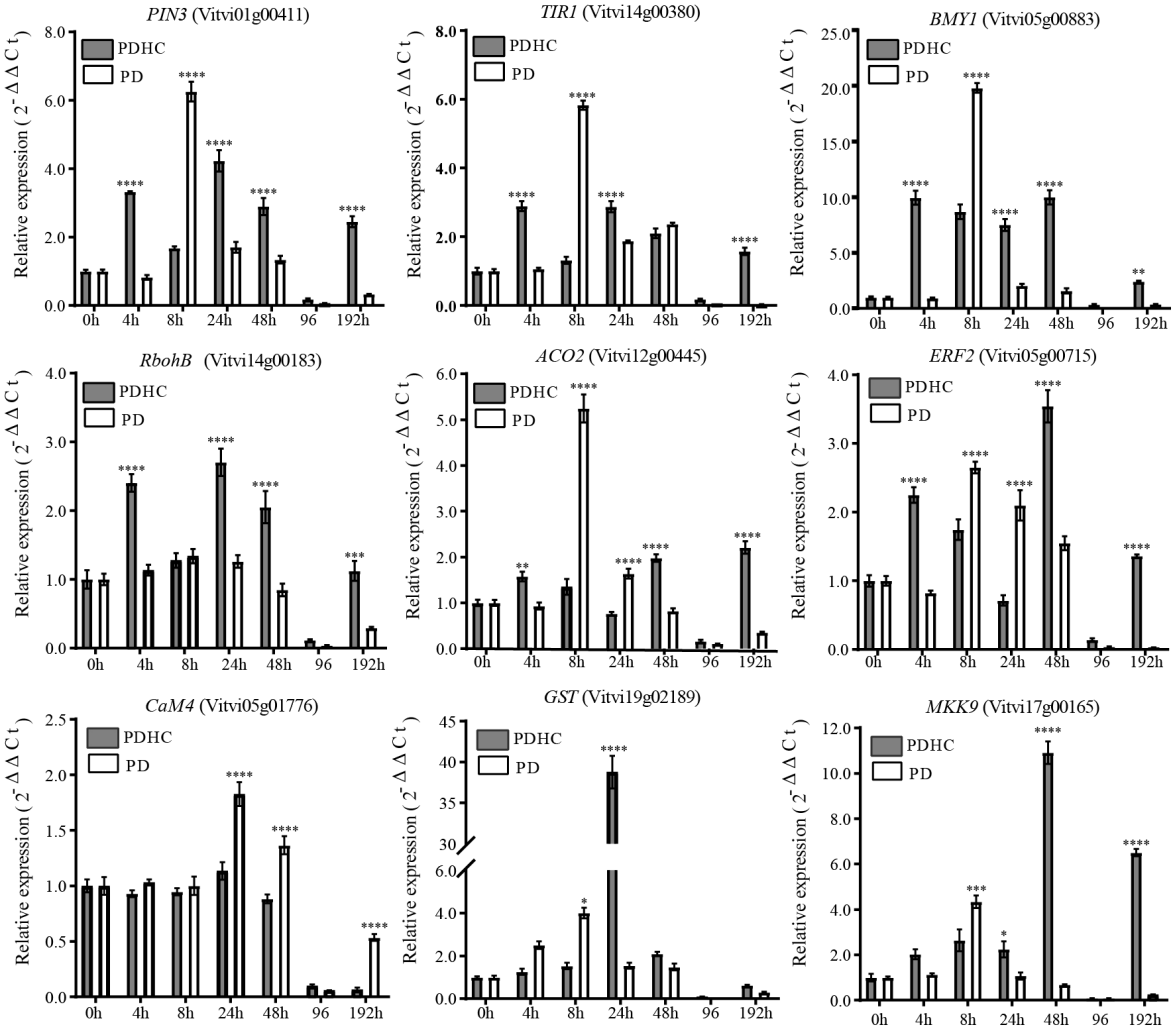
Comparation of transcript levels of signal transduction pathway-related genes between hydrogen cyanamide-treated group and control group. The data were analyzed using Student′s two-tailed unpaired t-test (*, *p*<0.05; **, *p* < 0.01; ***, *p*<0.001; **** *p <*0.0001).

To gain a better understanding of the effects of hydrogen cyanamide on improving bud sprouting rate and uniformity of paradormancy release, we conducted a more detailed time-course analysis using qRT-PCR to examine the expression patterns of genes related to signal transduction pathways. The results revealed that the expression trends of these genes were consistent with the transcriptome data. The expression levels of genes in the PD group were relatively higher than those in the PDHC treatment group, except for *VvGST*, *VvERF*, and *VvMKK9*. At 8 h after PD treatment, the expression levels of *VvPIN3*, *VvTIR1*, *VvBMY*, and *VvACO2* reached to highest point (**Figure 8**). The peak expression of *VvRbohB*, and *VvGST* occurred at 24 h after PDHC treatment, whereas the PD control group exhibited lower expression levels of these genes. Additionally, the genes *VvERF* and *VvMKK* exhibited high expression at 48 h after treatment. In comparison to the control group, the hydrogen cyanamide treatment induced these genes more rapidly and persistently, with expression levels higher than those of the control group from 24 to 48 h after treatment.

## Discussion

### The characteristic of paradormancy in grape winter bud

The results of single-node cutting experiments demonstrated that paradormant buds require approximately 7 days to be released from inhibition and undergo bud break, whereas endodormant buds require more than 30 days (Halaly et al., 2008; Perez and Noriega, 2018). In field conditions, the sprouting rate of paradormant buds reaches 50% within 7 days before June. However, as shoots undergo lignification and leaf senescence, the time required for bud break increases (He et al., 2012). Nevertheless, after the short pruning and leaf removal treatment, the sprouting rate reaches approximately 50% within a span of 14 days in August (Wei et al., 2022). The significant difference observed between leaf removal and retention subsequent to short pruning suggests that ABA, produced by senescence leaves, influences the sprouting of paradormant buds, although it is not the primary determinant of winter bud paradormancy (Perez and Noriega, 2018). Thus, it can be inferred that the inhibitory effects on bud growth primarily arise from apical dominance. Paradormancy represents an inhibited state rather than true dormancy (Dantas et al., 2020). Importantly, in second fruit cultivation, short pruning effectively induces the release of paradormancy.

### Comparative similarities in mechanisms of paradormancy and endodormancy release

**Figure 9:**
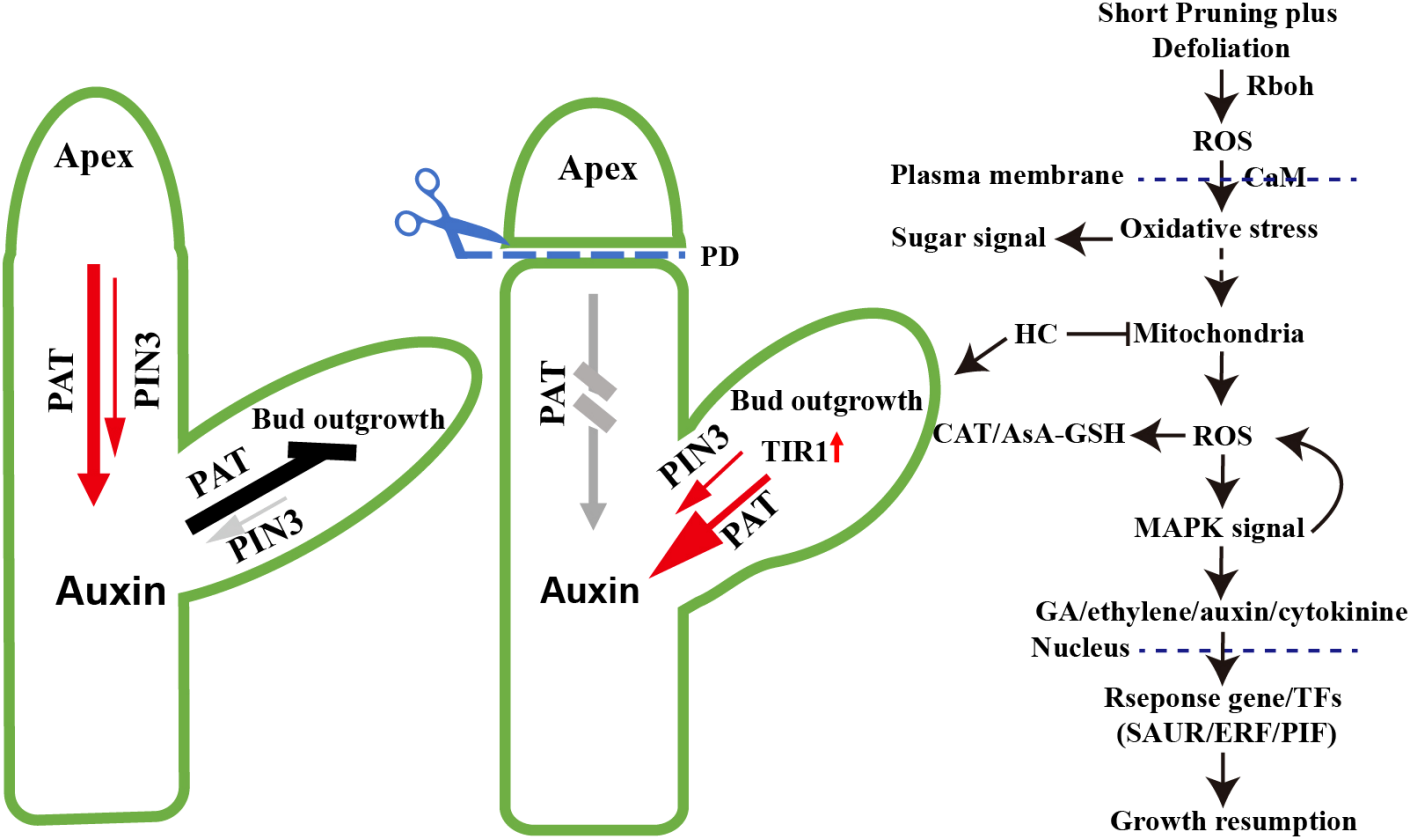
Model of paradormancy release hypothesis. According to the model, decapitation leads to the conversion of the sixth node winter bud into apical meristem tissue, resulting in the establishment of a polar auxin transport canal through the upregulated expression of *VvPIN3* and *VvTIR1*. This process leads to a reduction in auxin levels. Mechanical wounding caused by short pruning induces the production of extracellular ROS by Rboh. The ROS signaling facilitated by Calmodulin (CaM) crossing the plasma membrane, triggers respiratory stress and oxidative stress in the cytoplasm, leading to mitochondrial dysfunction and an increase in ROS production. This elevation in ROS levels activates the cellular antioxidant machinery, including the ascorbate-glutathione cycle (AsA-GSH) pathway and catalase system, which helps mitigate the oxidative burst. The MAPK signaling pathway is involved in the regulation of plant hormones such as GA, ethylene, auxin, and cytokinins, resulting in alterations in their levels. Consequently, response genes or transcription factors are activated, inducing continued growth of the winter bud meristem. In contrast, hydrogen cyanamide directly disrupts the mitochondrial electron transport chain (m-ETC), leading to ROS generation and establishing a more direct signaling pathway.

Based on transcriptome analysis, striking similarities were observed in the molecular regulation mechanisms between paradormancy release and previous studies on endodormancy release (**Figure 9**). Firstly, the processes involved in paradormancy release exhibit an overall downregulation of gene expression. Following 24 h of PDHC treatment, noticeable decrease was detected in the expression of cell cycle genes (*VvCYCD3*) and genes associated with DNA replication and repair. This downregulation pattern corresponds to the growth cessation observed during the release of endodormancy in natural conditions, reaching its lowest point during the deepest phase of endodormancy (Karlberg et al., 2010; Vergara et al., 2016; Dantas et al., 2020). This global change in gene expression can be interpreted as a preparatory phase for reactivating physiological activities and metabolic processes in anticipation of bud sprouting (Shi et al., 2020). Secondly, ROS play a crucial role as signaling molecules in the paradormancy release process. Treatment with PDHC induced mitochondrial dysfunction, leading to oxidative stress and temporary respiratory stress, which subsequently results in the generation of ROS (Takahashi et al., 2011; Vergara et al., 2012). As a response, the ascorbate-glutathione cycle pathway and catalase system are activated, as evidenced by the initial upregulation of gene expression of *VvGPX*, *VvGST*, *VvAPX*, and *VVCAT1* within 48 hours. These genes act as components of the active antioxidant machinery, and their expression levels decline at 96 hours (Sudawan et al., 2016). This pattern of gene expression is similar to that observed during endodormancy release (Ophir et al., 2009; Perez et al., 2009; Khalil-Ur-Rehman et al., 2019). Thirdly, ethylene is involved in the process of paradormancy release. There is an immediate upregulation of ethylene synthesis genes *VvACS* and *VvACO*, as well as an upregulation of *VvERF*. Ethylene has been found to participate in energy regeneration through the signaling gene *VvERF* during endodormancy release, where it acts as a significant antagonist of ABA, and VvERF serves as an energy-regenerating switch (Shi et al., 2018; Shi et al., 2020). Finally, the HC-induced stress response leads to a transient upregulation of gene expression levels related to starch and sucrose metabolism. Starch and sucrose metabolism play a critical role as a sugar source for energy, sugar signaling, and as components of cell walls, which are essential for growth recovery (Del Cueto et al., 2017; Wang et al., 2021). During paradormancy release, there is an upregulation of *VvBMY* expression, while in endodormancy release, the upregulation of *VvAMY1* and is induced (Ben Mohamed et al., 2010; Rubio et al., 2014). Additionally, genes associated with the trehalose synthesis pathway show upregulation, and the identification of trehalose further indicates its involvement in lateral bud growth. Additionally, upregulation of genes associated with the trehalose synthesis pathway has been observed, while trehalose has been confirmed plays a role in the lateral bud outgrowth (Mason et al., 2014; Fichtner et al., 2017).

### Comparative differences and positive role of hydrogen cyanamide in paradormancy release for second crop cultivation

The key distinction between paradormancy and endodormancy release lies in the removal of phytohormone inhibition. Paradormancy release involves the removal of the inhibitory effect of auxin on dormant buds, while endodormancy release involves the removal of the inhibitory effect of ABA on dormant buds (Zheng et al., 2015; Wang et al., 2021). After decapitation, the sixth node winter bud establish polar auxin transport canal by upregulated expression of the VvPIN3 and reduction in auxin levels (Balla et al., 2011). The elimination of ABA repression on meristem activity is achieved through the interplay between ethylene and ABA (Zheng et al., 2015). Secondly, the mechanisms of signal transduction differ between endodormancy release and paradormancy release. In the process of endodormancy release, hydrogen cyanamide treatment induce respiratory stress and leads to the production of H_2_O_2_ by disrupting the mitochondrial electron transport chain (m-ETC) (Perez et al., 2007; Ophir et al., 2009; Vergara et al., 2012). On the other hand, in paradormancy release, ROS production is induced directly by the CRK2, Rboh and MAPK signaling pathway (Kimura et al., 2020; Sozen et al., 2020). In the case of decapitation treatment, ROS is produced in the plasma membrane, and the MAPK signaling cascade amplifies the dormancy-breaking signals (Takahashi et al., 2011; Sozen et al., 2020). Adding HC treatment in paradormancy release can directly induce ROS production in mitochondria, thereby enhancing the dormancy-breaking signals (**Figure 9**). and HC treatment results in a longer induction efficacy of the growth hormone signaling pathway, and induces dormancy release and regulates growth recovery more quickly. This may be one of the reasons for the higher sprouting rate and improved uniformity observed in the hydrogen cyanamide-treated group.

In addition, the sprouting rate of paradormant buds is closely related to the accumulation of sugars and the nutritional conditions of the grapevines. Insufficient nutrient accumulation in the branches can impede bud break, while an abundant nutrient supply can promote rapid bud sprouting (Kebrom, 2017; Barbier et al., 2019). It is important to emphasize that the management during the growing season in second crop cultivation is more demanding. The entire fruit management period, from bud break to the second fruiting harvest, must be completed before the onset of annual low temperatures (Chen et al., 2017; Cheng et al., 2019). Irregular and patchy bud break often results in significant economic and viticultural challenges. Achieving high-speed and uniform paradormant bud break is crucial to meet these requirements. Hydrogen cyanamide treatment satisfies the production demands for uniform bud break, facilitating effective management. Therefore, the use of hydrogen cyanamide in production is reasonable. These conclusions contribute to our exploration of alternative stimuli agents.

## Data availability statement

The RNA-seq data presented in the study are deposited in the NCBI sequence read archive repository, accession number PRJNA1017535.

## Author contributions

GY and SF designed the research. SF, FL, YX, MW, and WC carried out experiments and collected and analyzed the data. SF wrote the manuscript. YX and FL revised the manuscript. All authors contributed to the article and approved the submitted version.
