# Supplementary figures and images for: Comparative transcriptome analysis of genes involved in paradormant bud release response in ‘Summer Black’ grape
Source: Front Plant Sci. 2023 Sep 25;14:1236141. doi: 10.3389/fpls.2023.1236141 (PMC10561283; doi:10.3389/fpls.2023.1236141)

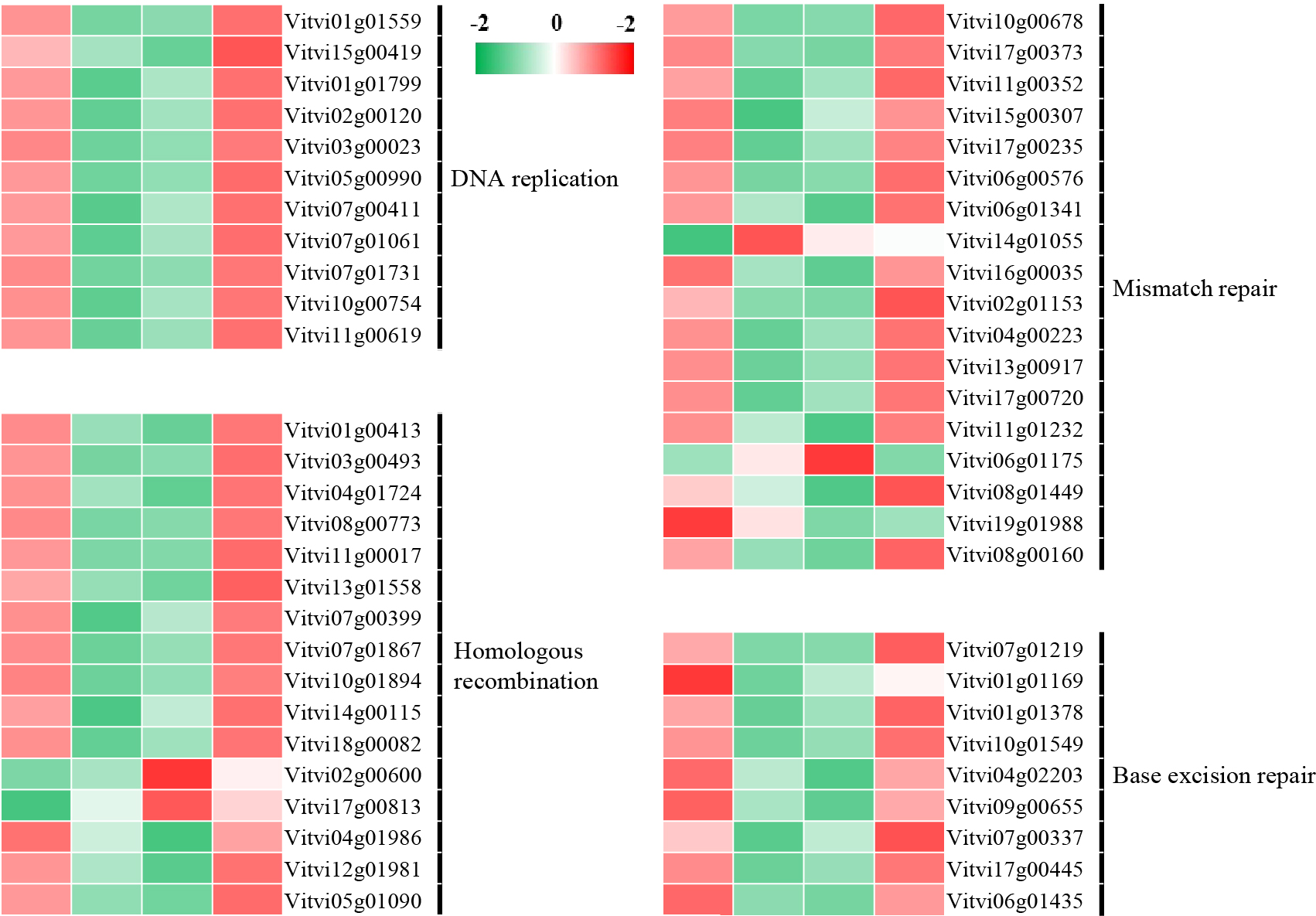

Supplement: Supplementary file 2 [file Image_2.jpeg]

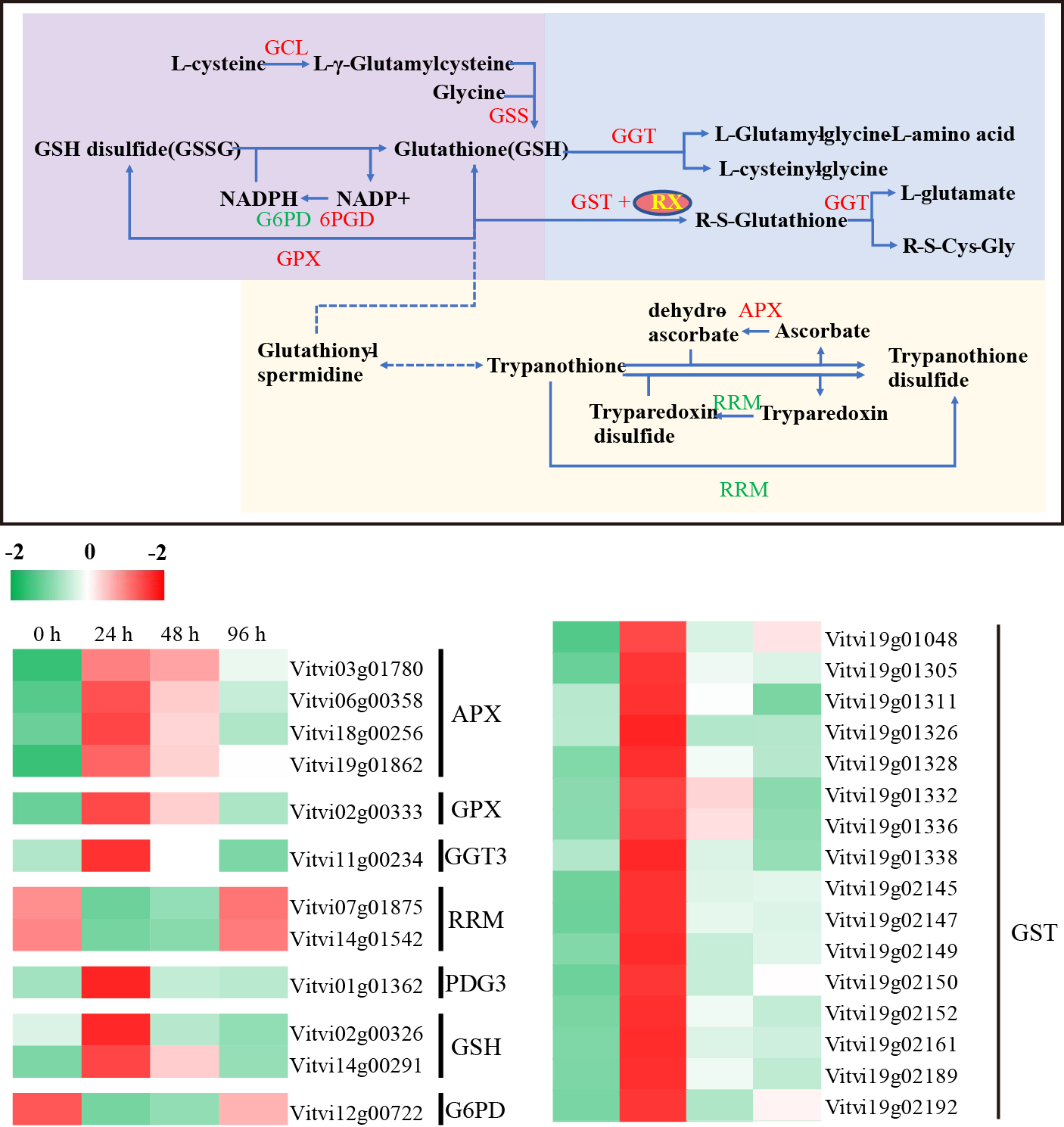

Supplement: Supplementary file 3 [file Image_3.jpeg]

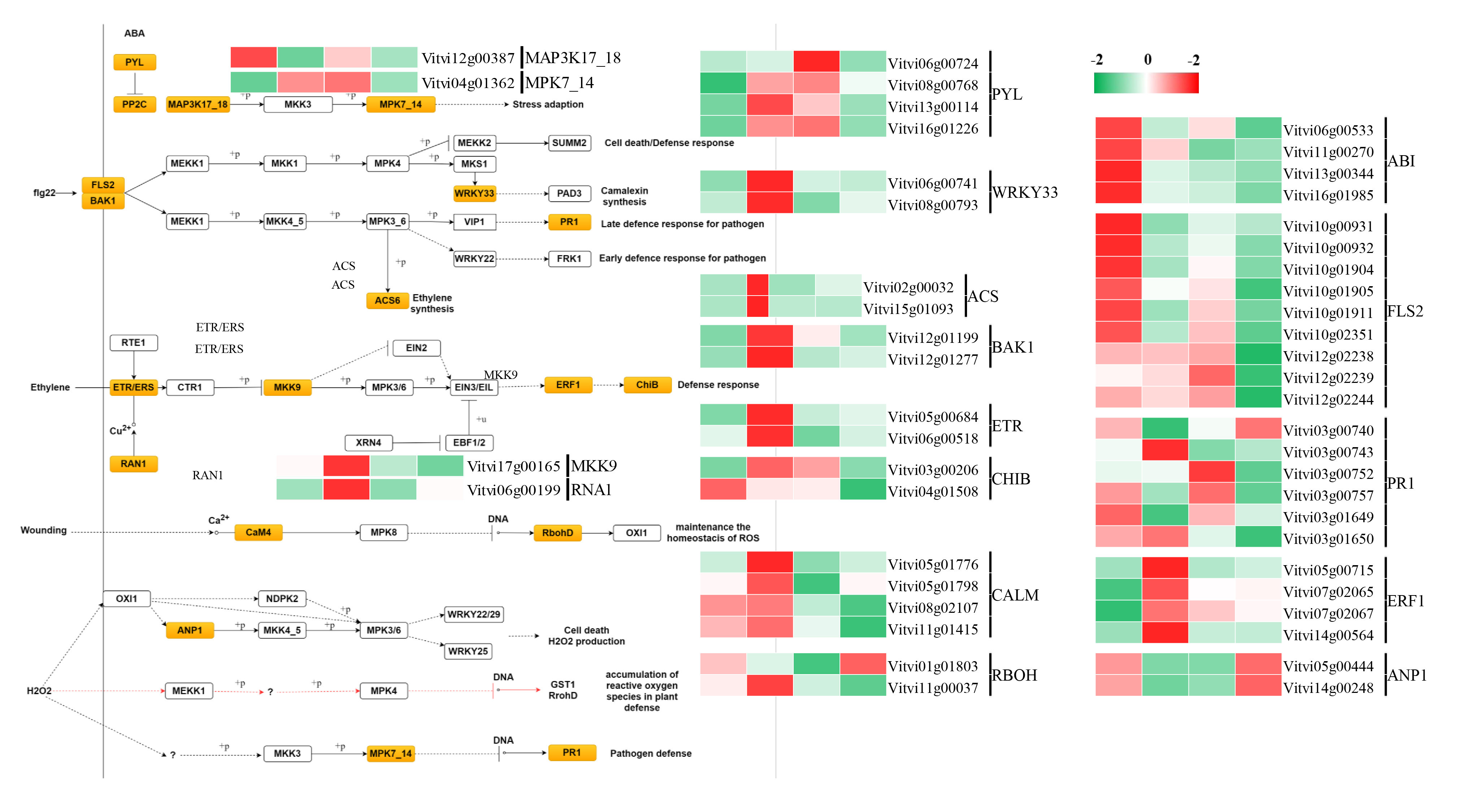

Supplement: Supplementary file 4 [file Image_4.jpeg]

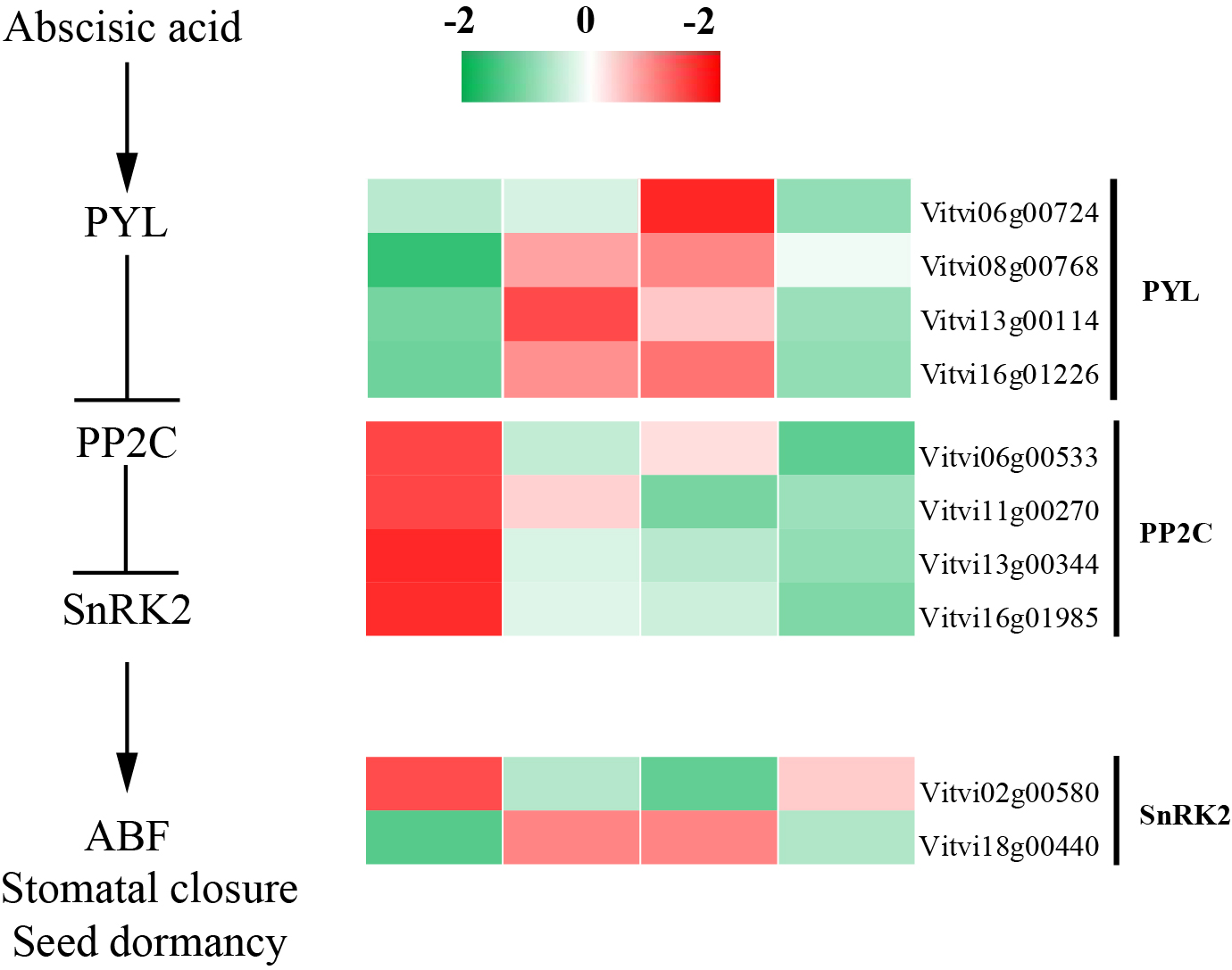

Supplement: Supplementary file 5 [file Image_5.jpeg]

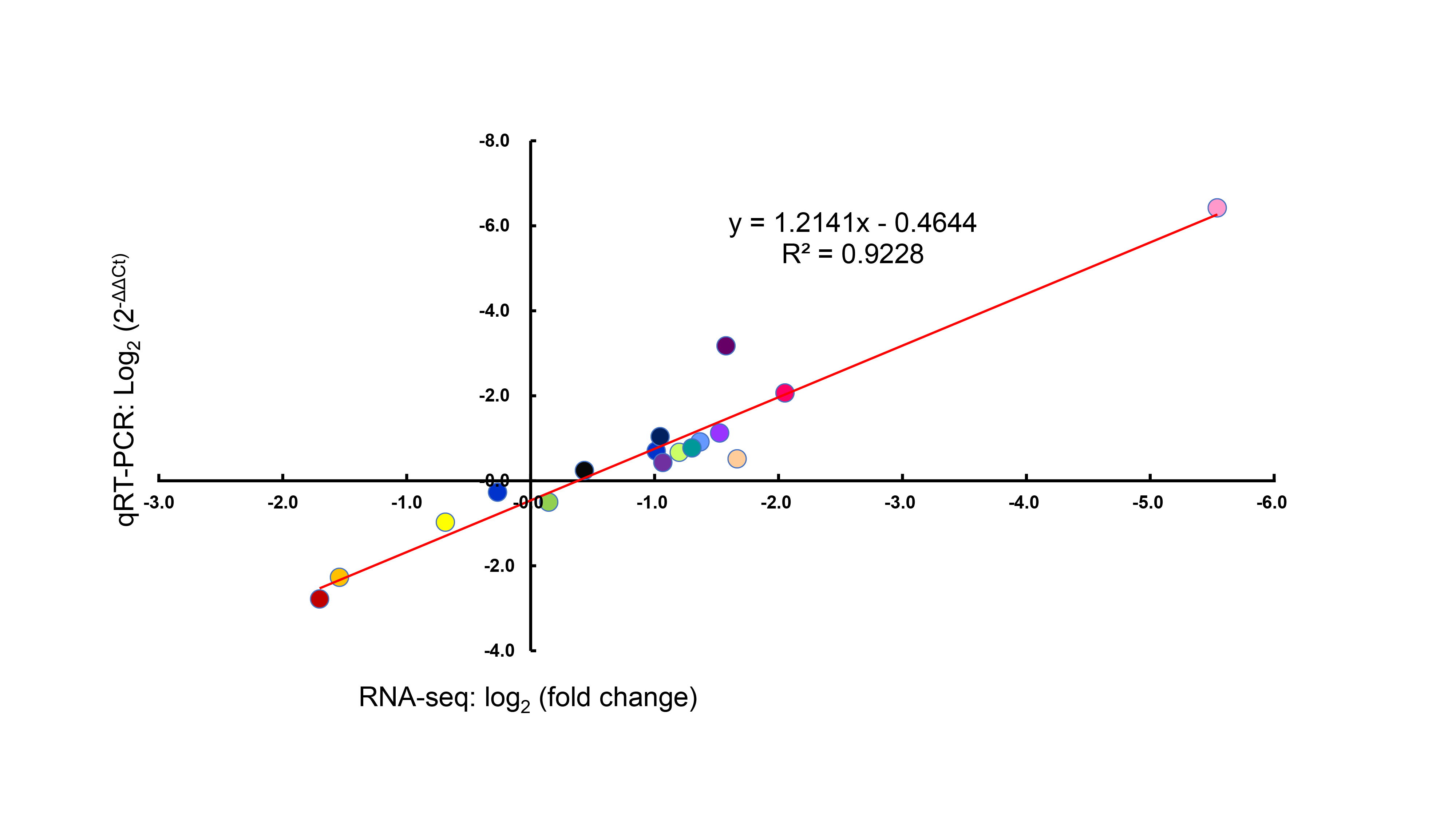

Supplement: Supplementary file 6 [file Image_6.jpeg]
